# Biological kinematics: a detailed review of the velocity-curvature power law calculation

**DOI:** 10.1007/s00221-025-07065-0

**Published:** 2025-04-03

**Authors:** Dagmar Scott Fraser, Massimiliano Di Luca, Jennifer Louise Cook

**Affiliations:** School of Psychology, University of Birmingham, Birmingham, UK

**Keywords:** Two-thirds power law, One-third power law, Kinematics, Noise, Filtering, Regression

## Abstract

The ‘one-third power law’, relating velocity to curvature is among the most established kinematic invariances in bodily movements. Despite being heralded amongst the ‘*kinematic laws of nature*’ (Flash 2021, p. 4), there is no consensus on its origin, common reporting practice, or vetted analytical protocol. Many legacy elements of analytical protocols in the literature are suboptimal, such as noise amplification from repeated differentiation, biases arising from filtering, log transformation distortion, and injudicious linear regression, all of which undermine power law calculations. Recent findings of power law divergences in clinical populations have highlighted the need for improved protocols. This article reviews prior power law calculation protocols, identifies suboptimal practices, before proposing candidate solutions grounded in the kinematics literature. We evaluate these candidates via two simple criteria: firstly, they must avoid spurious confirmation of the law, secondly, they must confirm the law when it is present. Ultimately, we synthesise candidate solutions into a vetted, modular protocol which we make freely available to the scientific community. The protocol’s modularity accommodates future analytical advances and permits re-use in broader kinematic science applications. We propose that adoption of this protocol will eliminate artificial confirmation of the law and facilitate more sensitive quantification of recently noted power law divergences, which are associated with neurochemical disturbances arising from dopaminergic drugs, and in conditions such as Parkinson’s and autism.

## Introduction

Binet and Courtier (1893) first reported that it takes longer to draw a tighter arc than a shallower arc of equal length (see also Jack 1895). It took almost one hundred years for these observations to be formalised empirically (Lacquaniti et al. 1983). Their formalisation encodes a negative relationship between curvature $\kappa$ and tangential velocity $v$—as curvature increases, velocity decreases—and that the exponent (i.e., power) of this empirically determined relationship is (minus) one-third. Hence, the one-third power law.

1 $$v \propto {\kappa }^{-1/3}$$

Lacquaniti and colleagues’ (1983) initial observation of the law, in 2-dimensional repetitive tracings of elliptical trajectories, has been widely replicated (Flash 2021; Huh and Sejnowski 2015; Matić and Gomez-Marin 2019; Richardson and Flash 2002; Viviani and Flash 1995; Zago et al. 2018; Zarandi et al. 2023) and extended to 3-dimensional space (Soechting and Terzuolo 1986; Massey et al. 1992; Sternad and Schaal 1999; Schaal and Sternad 2001; Flanders et al. 2006). The power law also extends beyond drawing movements to encompass foot movements (Ivanenko et al. 2002) and walked trajectories (Vieilledent et al. 2001; Hicheur et al. 2005; Pham et al. 2007). The law is observed in smooth pursuit eye movements (de’Sperati and Viviani 1997; Kowler et al. 2019), and tongue movements (Tasko and Westbury 2004; Perrier and Fuchs 2008; Kuberski and Gafos 2019). The power law is also found in non-human primate drawings (Schwartz 1994; Abeles et al. 2013), in the crawling of larvae (*Drosophila melanogaster*; Zago et al. 2016) and of the buff-tailed bumblebee (*Bombus terrestris*; James et al. 2020).

This ubiquity of the power law in motor production—gross, fine and across species—has led to suggestions that it is a law underpinning all biological movement. Harris and Wolpert (1998) demonstrate the power law explains disparate human movements of eye and arm, whilst Zago and colleagues (2016) find this ‘*fundamental law of human control*’ in the movements of larvae, for Omrani and colleagues (2017) it is one of the ‘*regularities in biological motion*’, Flash (2021, p. 4) declare it as one of the ‘*kinematic laws of nature*’, Torricelli and colleagues (2022) present it as a ‘*motor invariance*’ par excellence, and Frith and Frith (2023, p. 100) offer it as one of the ‘*kinematic regularities*’ that observers use to predict biological motion. Indeed, the literature has leveraged the power law to produce synthetic ‘*biological*’ motion, to enhance familiarity in robot-human dyads (Hugues et al. 2016; Maurice et al. 2018; West et al. 2022; Edraki et al. 2023; Chen et al. 2024) and in teleoperation (Rybarczyk and Carvalho 2017).

The mechanism by which the power law emerges remains unknown. Lacquaniti and colleagues (1983) propose the power law is neural; *‘a built-in tendency of the motor control system’.* This could arise as a neural (i.e., motor planning) schema, such as one which maximises smoothness by minimising jerk (Viviani and Flash 1995; Pham et al. 2007; Huh and Sejnowski 2015; Boulanger et al. 2024), or minimising endpoint variance in the presence of noise (Harris and Wolpert 1998). Movement segments, obeying the power law, have been shown to exhibit constant equi-affine velocity (Pollick and Sapiro 1997; Langlois et al. 2024) which is a candidate for shared motor perception and motor production neural coding (Flash and Handzel 2007). Others speculate that the law may be biomechanically embodied; driven by the noise inherent in the motor system (Maoz et al. 2005) or as a by-product of the low pass filtering effects of the musculo-skeletal system (Gribble and Ostry 1996; Schaal and Sternad 2001; Matić 2022). A third perspective is that the power law is a statistical illusion (Marken and Shaffer 2017).

## Current trends in power law research

A review of methods for calculating the power law is especially timely, as the field is energised by the increasing availability of means for capturing human movement, escaping the restrictions of laboratory-based apparatus. The field has moved from the Edison pen of the 1890s, to electronic drafting tables of the early 1980s and 1990s, to instrumented professional drawing surfaces (such as those made by WACOM) in the 2000s, to widely available consumer tablets such as the iPad launched in 2010. Each advance increases availability and convenience, transitioning from niche professional to widely available consumer devices. A recent open-source application for Android tablets, with accompanying Python analysis protocol (Matić and Gomez-Marin 2019) has made power law calculation easily accessible using consumer tablet devices. The trend continues with recent work (Gupta et al. 2023), utilising smartwatch accelerometers measurements to derive the power law. Given the advances in hand tracking and the characterisation of virtual reality headsets as research platforms (Abdlkarim et al. 2024), we anticipate power law research will follow.

**Fig. 1:**
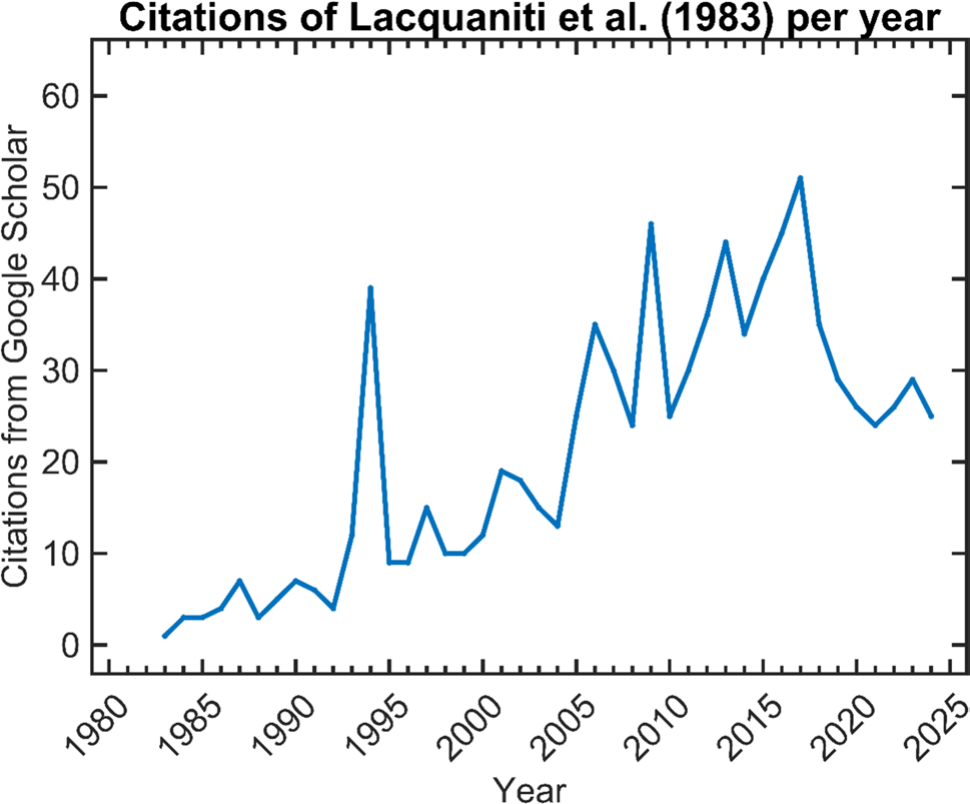
Papers per year, citing Lacquaniti et al. (1983), as recovered from Google scholar, up to year ending 2024

In the 40 + years since the power law’s formalisation 883 articles (Google Scholar, published 2024 or earlier) have cited Lacquaniti and colleagues (1983). A simple yearly average of ~ 22 citations place it in the top twenty-five experimental psychology papers of its era (Appendix A, 1980–1984; Cho et al. 2012). The last ten years has seen it cited 330 times, at 1.85 times the rate of the previous thirty years, showing accelerating interest (Fig. 1).

Interest has been further invigorated by suggestions that subtle divergences from the power law may serve as indicators of clinical conditions and neurochemical disturbances. Recent studies (Lu et al. 2023; Cook et al. 2023; Fourie et al. 2024) have reported divergences from the power law one-third value in drawings made by autistic people. Hickman and colleagues (2024) show power law deviation in dopaminergically challenged populations—e.g., Parkinson’s patients before medication and in neurotypical individuals administered haloperidol, a dopamine D2 receptor antagonist. James and colleagues (2020) report that the movements of the buff tailed bumblebee vary from the one-third power law when exposed to neonicotinoid pesticides. At present, since the origins of the power law are opaque, it is unclear whether these divergences from the law arise due to differences in neural codes, changes to biomechanical constraints, or indeed statistical artefact (Marken and Shaffer 2017). Studies of these power law divergences pave the way for applications that can detect clinical conditions and neurochemical disruptions based on the succinct result of the power law exponent calculation. Such applications could have widespread utility across both clinical and non-clinical settings. However, such uses demand increased sensitivity from methodological and analytical choices, when detection relies on classification using a singular resulting value. The power law is also deployed to create predictable ‘*biological*’ motion for robot-human dyads (Hugues et al. 2016; Maurice et al. 2018; West et al. 2022; Edraki et al. 2023; Chen et al. 2024), and for enhancing robot teleoperation (Rybarczyk and Carvalho 2017), scenarios which could benefit from refinement of the law’s approximate one-third value.

## Issues with power law precision and reporting

Major issues in achieving precision in power law calculation arise at least in part, due to differing methodologies, the lack of vetted analytical practices or common reporting practices in this field. Karklinsky and Flash (2015) note that differing strategies to calculate the power law’s exponent offer varying results, and need to be systematically explored. This is not unique to power law analysis, often methodological and analysis choices lead to differing results from the same data (Gelman and Loken 2014; Kummerfeld and Jones 2023). What is unique with power law analysis is that issues with common power law analytical choices (of differentiation, log projection, regression, and/or filtering) may individually, or in concert, bias results to artificially confirm the approximate one-third value. At best, such bias obfuscates intriguing and potentially diagnostically useful divergences, at worst it may drive the ubiquity of the law. Any candidate fundamental law of biological movement should be underwritten by a rigorous, vetted protocol for its calculation. Matić and Gomez-Marin (Matić and Gomez-Marin 2019) have already provided a power law calculation package. However, this prior implementation includes multiple ‘*legacy*’ analytical steps that have potentially been included because they are common, not necessarily best, practice i.e., Butterworth filtering, finite differences differentiation and log projected linear regression. Such steps may bias the power law calculations. In the body of the review, these individual legacy steps will be compared theoretically and numerically against candidate best practices drawn from the literature, with professional MATLAB implementations, before concluding which should be considered vetted and adopted for future work. The rigour of the ‘vetted’ practices will be demonstrated by meeting two criteria; firstly, rejection of spurious one-third power law where it can be analytically rejected, secondly recovery of the one-third power law where is can be expected analytically. These vetted practices are then shared in an open science implementation. These crucial steps build toward common methodological and analytical practices, which will allow results to be compared confidently between papers, and divergences from the power law to be better quantified and exploited.

## Legacy approaches to velocity-curvature power law calculation

Contemporary calculation of power law values largely conforms to the methods outlined by Lacquaniti and colleagues (1983). In this section we outline this ‘*legacy*’ approach to power law calculation. To obtain the empirical values of the power law, first recover coordinates in $x$ and $y$ at time $t$, from some curvilinear drawing task. Then smooth these trajectories to remove noise, such as by passing them through a 2^nd^ order, 10 Hz low pass, zero lag Butterworth filter. Tangential velocity may then be calculated given the changes in $x$ and $y$ (Viviani and Stucchi 1992; Viviani and Flash 1995; Gribble and Ostry 1996; Maoz et al. 2005; Matić and Gomez-Marin 2019) using this classic formulation;

2 $$v={( {dx}^{2} + {dy}^{2})}^{1/2}$$

Curvature is the reciprocal of the radius of curvature (i.e., $1/r$). Where $r$ is the radius of the ‘*best fitting circle*’ (i.e., the osculating circle) which is tangential to the trajectory at time $t$. This may be calculated directly from velocity and acceleration (O’Neill 1966; Viviani and Stucchi 1992);

3 $$\kappa = \frac{{\left| {\left( {dx)(d^{2} y} \right) {-} (d^{2} x)\left( {dy} \right)} \right|}}{{\left( { dx^{2} + dy^{2} } \right)^{3/2} }}$$

**Fig. 2:**
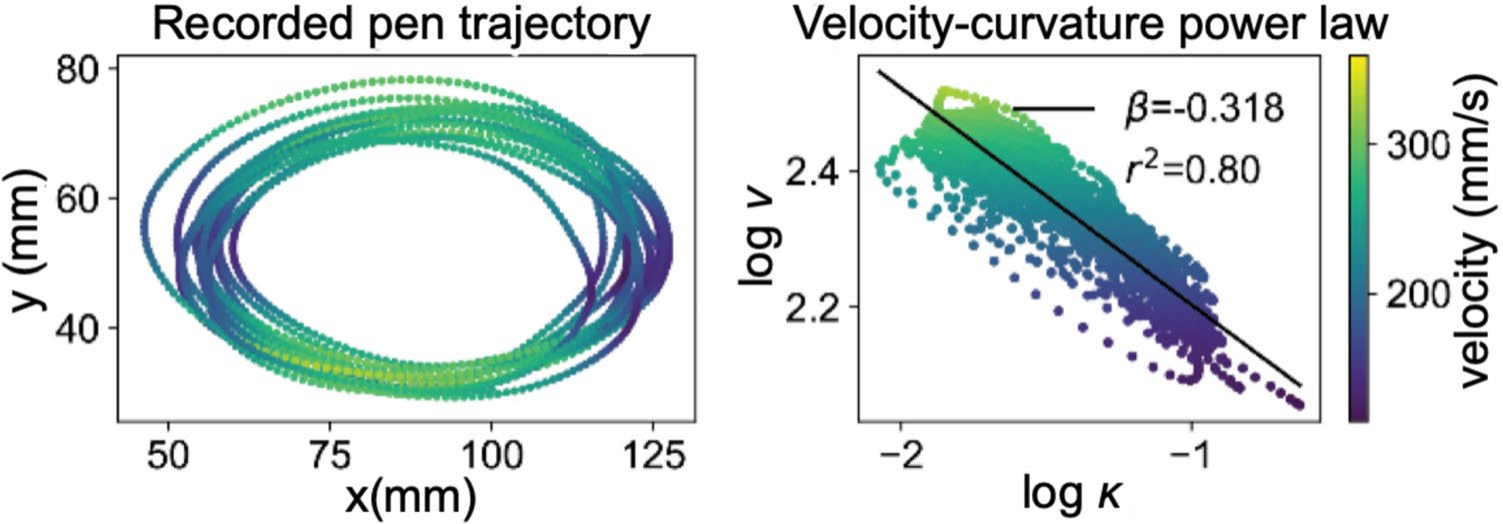
The classic ellipse demonstrating the roughly ‘*one-third*’ power law (Matić 2022). The leftmost panel shows pen coordinates in *x* and *y* recovered in millimetres, across time course *t*. The rightmost panel shows the log projected tangential velocity and curvature, linearly regressed to give a *β* of roughly minus one-third. Velocity gain factor is not reported. Adapted and used with permission (Matić 2022)

The tangential velocity $v$ and curvature $\kappa$ are logarithmically projected, as shown below in Fig. 2, allowing Eq. (4) to be recovered empirically. To recover the relationship simple linear regression is employed giving *β* as the signature slope, and a necessarily constant y-axis intercept, the log of the velocity gain factor (VGF)*.*

4 $${\text{log}}v = {\text{log}}VGF - \beta {\text{log}}\kappa$$

Reversing the log-transformation reveals the classic power law form (Eq. 4), in which *β* is the one-third power law exponent (as previously introduced in a simplified form in Eq. 1);

5 $$v = VGF \, \kappa^{ - \beta }$$

Thus, the tangential velocity (*v*) is equal to the VGF*,*^1^ multiplied by curvature (*κ*), to the negative power of the curvature exponent (*β* i.e., the ‘*power law exponent*’). In this, the tangential velocity form of the power law, it has been found empirically that *β* is roughly one-third. Hence the ‘*one-third power law*’. An example of such is found in Fig. 2.

## Alternate power law formulation

**Fig. 3:**
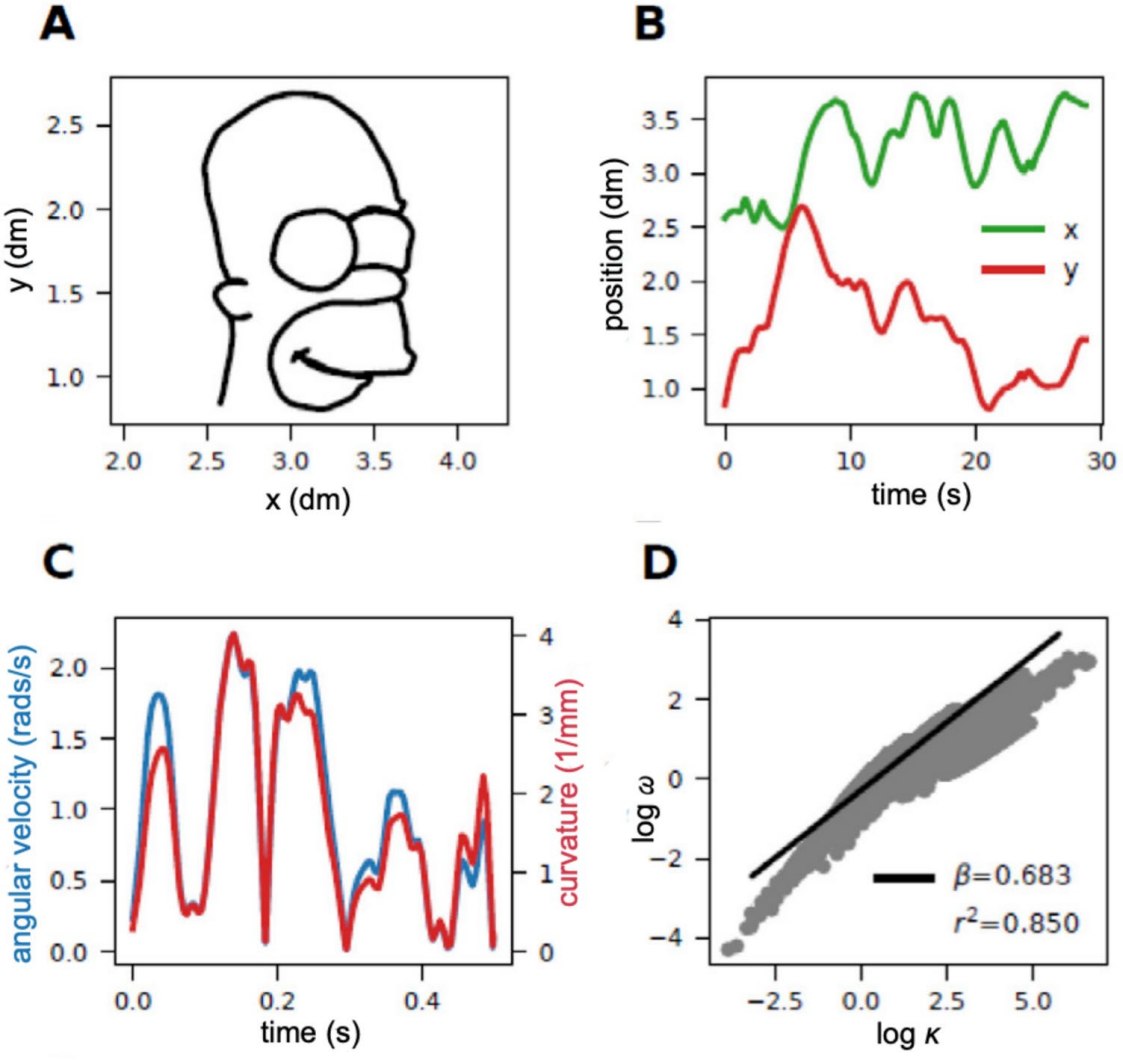
Frame A shows a doodle in *x* and *y* in decimetres. Frame B shows *x* and *y* across time course *t*. Frame C shows the angular velocity and curvature across *t*. Frame D shows the log–log projection of the values from frame C plotted against each other, and then linearly regressed to give rise to an approximately two-thirds’ power law *β* exponent. Note no report of velocity gain factor. Adapted and used with permission (Matic and Gomez-Marin 2019)

A second formulation of the power law was initially presented in the foundational article (Lacquaniti et al. 1983), and whilst rarer, still appears in the literature (e.g., James et al. 2020). We may also express a power law for angular velocity ω (measured in radians per second), which is related to tangential velocity *v* (a measure in metres per second) via *ω* = *r* × *v*. This reformulates the power law as so:

Where the angular velocity (ω) is expressed as the multiplication of the VGF, times the radius of curvature (*r),* to the negative power of the curvature exponent (*β)*. In this angular velocity form of the power law, *β* empirically takes the value of roughly two-thirds, hence the more common name of ‘*two-thirds power law*’. We present an example of this in Fig. 3. This form suffers from infinite radius at linear inflection points, unlike Eq. (5)’s curvature which may be taken as zero at these points (though technically $\frac{1}{\infty }$ is undefined), which explains the alternative one-third formulation’s pre-eminence.

These equivalent tangential and angular velocity forms of the power law, in Eqs. (5) and (6), are both introduced in the foundational paper (Lacquaniti et al. 1983). Here we adopt the one-third formulation throughout, reporting values from the literature after conversion where necessary (i.e., subtract 1 from *β* for Eq. 6).

## Considering deviations from the one-third power law

Power law research must be tempered with critical voices that ultimately call into question the very existence of the power law. Critical voices (Wann et al. 1988; Sternad and Schaal 1999; Schaal and Sternad 2001) question the existence of the one-third power law, highlighting that observed values do not always strictly adhere to the headline one-third value (even in typical cohorts). These voices, at least implicitly, argue that a so called ‘*fundamental law*’ should have a single, fundamental value. Although studies of the power law in non-human species, including larvae (Zago et al. 2016) and elephants (Dagenais et al. 2021), are easily interpreted as evidence of the cross-species universality of the power law, more careful examination of these studies reveals power law exponent values that deviate from the one-third value. Although the power law found in the unconstrained movement of elephants’ trunks has been referred to as being *‘similar to human hand drawing’,* it has a divergent value of two-thirds (Dagenais et al. 2021). Similarly, although the power law noted in the movement of larvae has been referred to as the ‘*fundamental*’ power law, its actual value approaches one-quarter, rather than one-third (Zago et al. 2016). In human tongue movements the value of the power law exponent is variable, or conforms to one-third only in higher frequencies of utterances (Kuberski and Gafos 2019). Thus, multiple studies find deviations from the one-third value.

**Fig. 4:**
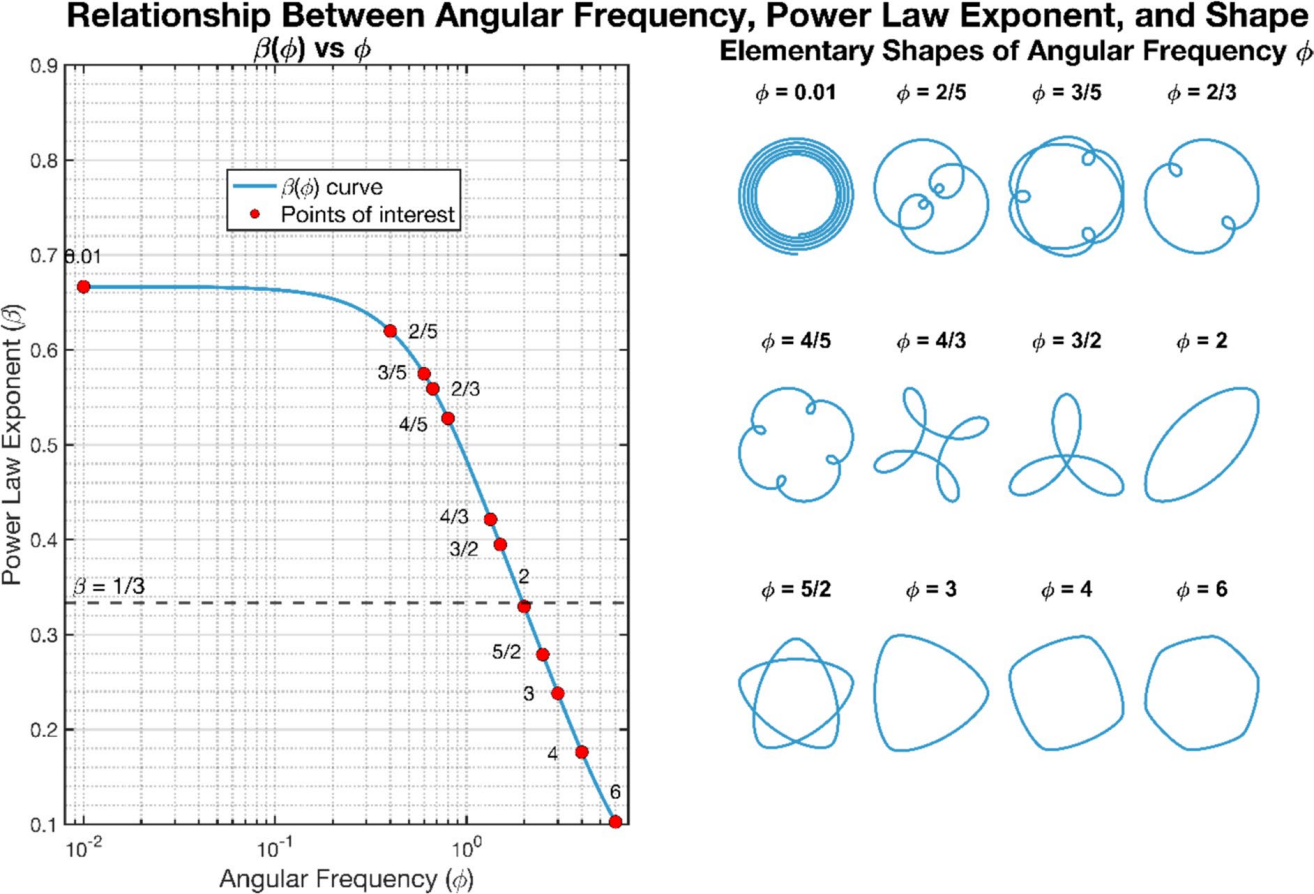
(Left panel) A spectrum of power laws, calculated via analytical formula, derived within a minimum-jerk optimal framework (Huh and Sejnowski 2015) in which the ellipse with angular frequency *φ* of two, gives rise to the one-third value. (Right panel) Angular frequency *φ* is defined as the number of curvature oscillations per 360° cycle of angular displacement around a given shape i.e. how frequently the curvature value of a path varies as you move along the shape’s perimeter. A circle has angular frequency zero as the curvature is constant. An ellipse has angular frequency two, because in every cycle there are two curvature oscillations i.e., from near flat to a tight corner twice in 360°. A rounded square has an angular frequency of four corresponding to four oscillations in curvature. A logarithmic spiral has an angular frequency approaching zero, because in every 360° cycle of angular displacement there is only a small (near zero) change in trajectory curvature i.e., it is near circular

Deviations from this one-third value need not be fatal to the power law. In a notable study Huh and Sejnowski (2015) revealed that the power law exponent *β* is not a universal constant, but that divergent values emerge naturally from the optimisation of movement smoothness. By applying the minimum jerk model to a range of movement trajectories, they analytically derived a formula that predicts how the power law exponent varies with the angular frequency *φ* of different trajectory shapes (see Fig. 4). We direct readers to the original paper for the complete analytical formula. This approach resulted in a ‘*spectrum of power laws*’—a continuous range of power law exponents that are all consistent with the same fundamental principle of movement smoothness. For elliptical trajectories (*φ* = 2), Huh and Sejnowski’s model predicts *β* = 0.330, closely matching the classical one-third value. Shapes with lower angular frequencies (like spirals) exhibit higher exponents, and shapes with higher angular frequencies (like rounded polygons) show lower exponents. Huh and Sejnowski verified their theoretical prediction through empirical measurements. The spectrum was reproduced by Zago and colleagues (2018), confirming that the minimum jerk principle can account for the systematic variations in power law exponents observed across different movement patterns. Therefore, we may entertain that elephants may draw segments of logarithmic spirals with their trunk tips (Dagenais et al. 2021). In this case the trunk’s empirical power law exponent would align with the predicted value of *φ* = 0.01 from the spectrum of power laws.

Huh and Sejnowski further show that combinations of angular frequency shapes, such as an elliptical spiral, exhibit a value that is a weighted combination of their component power laws. As such an arbitrary power law exponent (within the range covered by the spectrum) could be generated with a suitable combination of shapes. Thus, to assess power law exponents in non-elliptical movement it is essential to account for the movement trajectory and consider the predictions of the spectrum of power laws formula of Huh and Sejnowski.

This spectrum could account for freehand drawings trajectories’ one-third power law, which could arise from a weighted combination of the shapes in Fig. 4’s right panel. However, trajectories can be expressed as a series of elliptical motifs (Dhieb et al. 2022). Indeed, any planar ellipse is merely two coupled orthogonal sinusoid and co-sinusoids of equal frequency. More generally, Lissajous (or Bowditch) curves, which are coupled sinusoids of arbitrary frequency and phase difference (of which the ellipse is therefore a special case) have been shown analytically and empirically to exhibit the one third power law (Viviani and Schneider 1991). As two coupled sinusoids can model strokes in a continuous drawing task (Hollerbach 1981; André et al. 2014), this combination of elliptical motifs may be parsimonious, not requiring the combined shapes of Huh and Sejnowski. However muscular generation of pure sinusoids has attracted criticism (Wann et al. 1988).

## Analytical issues concerning noise, differentiation, and filtering

Each step in the legacy analysis protocol outlined previously (see Legacy approaches to velocity-curvature power law calculation) may affect the reliability or even bias the result of power law (*β*) calculations. Caution in power law calculation is warranted as velocity, acceleration and in turn curvature derive from differentiated coordinates (Eqs. 2 and 5, e.g., Viviani and Stucchi 1992; Matić and Gomez-Marin 2019). Commonly employed finite differences differentiation (e.g., MATLAB *diff* function), compounds noise power in each successive step i.e., coordinates to velocity, and thence to acceleration (Pezzack et al. 1977; Chartrand 2011; van Breugel et al. 2020). This issue is especially concerning as the power law calculation has been demonstrated to be specifically sensitive to noise. Maoz and colleagues (2005) demonstrated that random walks (trajectories with *x* and *y* coordinates independently drawn from Gaussian white noise and assigned sequential time points *t*) demonstrate power law compliance when analysed using legacy protocol elements (i.e., finite differences differentiation, log–log projection and linear filtering). Similarly, when they simulated constant tangential velocity (*β* = 0, see Eq. 10) elliptical trajectories (100 mm major, 50 mm minor axis at 100 Hz) with added 2 mm standard deviation Gaussian white noise (of a similar magnitude to the minimum average error found in those undertaking a shape drawing task in Madirolas et al. 2022), they also found the artificial emergence of an approximately one-third power law, when analysed with legacy protocol elements. This effect was so pronounced it led to suggestions the power law may arise from motor noise itself (Maoz et al. 2005). It follows that any type of noise e.g., motor noise, but additionally sensor and quantisation noise (i.e., difference between true signal and digitisation value) may bias power law exponents. Thus, maximising signal to noise ratio (SNR) in any recording strategy is recommended to tease apart intrinsic noise from measurement noise.

Digital filtering is a common step to maximise SNR, however filtering alters the underlying signal, and may still admit noise (Maoz et al. 2005; Chartrand 2011; van Breugel et al. 2020). Further, the power law may very well originate in the biomechanical low pass filtering effect of joints on motion (Gribble and Ostry 1996; Schaal and Sternad 2001; Matić 2022). As such, given the similarity of effect of digital filters to a joint’s filtering effect on derived kinematic values, the use of digital filters may offer spurious evidence of biomechanical filtering effects in power law analysis. Therefore, special attention must be paid when employing filters. For example, passing a non-law compliant elliptical trajectory generated with *β* = 0 rather than one-third (i.e., constant tangential velocity motion) through a 4^th^ order low pass Butterworth filter gives rise to a spurious power law exponent of one-third (Schaal and Sternad 2001).^2^ This finding of Schaal and Sternad is concerning, as common denoising filters employed to pre-process kinematic data are in effect 4^th^ order. The classic ‘*zero lag’* low pass 2^nd^ order Butterworth filter used in power law analysis (e.g., Zago et al. 2016; Matić and Gomez-Marin 2019; Zarandi et al. 2023) obtains zero lag by running twice. Once forward, and once back to address lag, i.e., temporal shifts in filtered data event timings introduced by running forwards (de Cheveigné and Nelken 2019). Thus, zero lag ‘2^nd^’ order filters are effectively 4^th^ order filters and may bias power law values.

Given the power law calculation’s sensitivity to filtering noted above, additional hidden filtering and smoothing steps may mask or distort the power law calculations, and as such should be avoided. For example; splining the raw data is another common pre-processing step (Schaal and Sternad 2001; Matić and Gomez-Marin 2019), however this alters the trajectory in a fashion akin to a low pass filter, by smoothing the data.

In sum, although filtering and splining to maximise SNR are commonly employed, caution must be used lest they bias power law values. It can thus be argued that prior one-third power law calculations that have employed finite differences, and/or common filtering strategies may be biased. In some cases, the resultant power law values could even be described as statistical artefacts of noise, and/or filtering. Given differentiation and filtering have undesirable and compounding impacts, alternate methods which reduce the number of calculation steps between coordinates and denoised velocity/acceleration are desirable.

## Candidate analytical steps addressing noise, differentiation, and filtering

An alternative which permits filtering and differentiation parsimoniously, is the differential filter or smoothing differentiator. An example of this is the Savitzky-Golay (SG) filter; offering zero lag denoising and differentiation (to any arbitrary level) in a single step (Savitzky and Golay 1964). This avoids the compounding noise power of successive finite differences differentiation steps, and potentially the necessity for aggressive filtering. SG filters are simple to implement, with two parameters, window length and order, as with the Butterworth filter’s corner frequency and order (though note zero lag as a hidden third parameter). Higher order Butterworth filters smooth more powerfully than low order, whereas lower order SG smooth more powerfully than higher. Whilst there is no consensus on these SG parameters for human kinematics, unlike the ubiquitous Butterworth parameters; low pass corner frequency ~ 10 Hz, 2^nd^ order, zero lag (therefore 4^th^ order), Crenna and colleagues (2021) designed a SG filter (width seventeen, fourth order), specifically to emulate the 10 Hz lowpass Butterworth. Caution must be exercised as lower order SGs may potentially replicate the observed biasing effects of the higher order Butterworth filters. Therefore power law exponents should be demonstrated using a range of filter designs (Zago et al. 2016; 2018) drawn from a principled choice of SG parameters (Luo et al. 2005; Vivó-Truyols and Schoenmakers 2006). In all cases the denoising filters should offer unity gain. Unity gain entails that the underlying signal is not inflated or diminished, which would bias the y axis intercept, i.e., the VGF, in any ensuing regression. This also allows the true to life report of derived values such as velocity and acceleration, which would otherwise be altered by non-unity gain filters. SG can also be applied to alternative analyses that allow curvature and tangential velocity to be recovered by transforming trajectories into the Frenet-Serret frame, as these still include multiple differentiation steps (Huh and Sejnowski 2015; Dagenais et al. 2021; Cook et al. 2023; Hickman et al. 2024).

The number of required differentiation steps could be, in theory, reduced by implementation of geometric curvature as opposed to the kinematic curvature of Eq. (3), however there are some practical limitations to this approach. Menger’s curvature offers a formula by which three trajectory points, forming a triangle, can give rise to curvature at the middle point (Menger 1930). However, such geometric curvature calculations are sensitive to noise and exhibit a lack of numerical stability when a curve is oversampled i.e., successive values appear co-linear, and so the triangle is degenerate. This instability arises as a function of machine precision, sampling rate and local geometry (Deshmukh et al. 2020). Varying the sampling rate can reveal oversampling but further research is required, comparing geometric and kinematic calculations. We therefore cannot recommend adopting Menger curvature approaches at this point.

To summarise, we note that common steps in the legacy analysis protocol outlined previously may affect the reliability or even bias the value of power law calculations. We propose the SG filter as a candidate element of the vetted protocol, to replace legacy finite differences differentiation and Butterworth filtering. We implement such a differential filter below (see Candidate velocity-curvature power law protocol implementation).

## Impact of rival formulations of the velocity-curvature power law

The formulations of the power law, based on curvature and either angular or tangential velocity, seen in Eqs. (5) and (6), are formally equivalent (Lacquaniti et al. 1983). Whilst theoretically true, it has been empirically demonstrated to be unsound in the presence of noise (Matić and Gomez-Marin 2022). Note Eq. (6)’s angular velocity $\omega$ may be re-expressed as$v \times r$, where $r$ is radius of curvature. Substituting this into Eq. (6) ($\omega$ $= VGF {r}^{-\beta } )$ and taking the log transformation gives;

9 $$\text{log}v+ \text{log}r= \text{log}VGF- \beta \text{log}r$$

Each side of Eq. (9) has the correlated term log *r*. This is problematic in two related ways. First, calculating radius of curvature r is inherently noisy. When the trajectory becomes straighter or passes through inflection points, the radius approaches infinity, meaning very small measurement errors in position can lead to very large errors in radius calculations, which is then compounded due to the computer’s inability to precisely represent these extreme values (i.e., default machine precision in MATLAB is 16 digits). Secondly, because log *r* appears on both sides of Eq. (9), any noise in the radius measurement affects both the dependent and independent variables in a correlated way. If a noisy radius measurement is too large, it increases both log *v* + log *r* (left side) and log *r* (right side) simultaneously. Similarly, if the radius measurement is too small, it decreases both sides together. This correlated movement creates an artificial pattern that makes the relationship appear stronger than it really is, leading to inflated goodness-of-fit values in angular velocity power law linear regressions (Matić and Gomez-Marin 2022).

In contrast, the tangential velocity formulation (Eq. 5) expresses the relationship using curvature (κ) and tangential velocity (*v*). While both are derived from the same velocity components (Eqs. 2 and 3), their relationship to these components differs—velocity has a direct relationship through first derivatives, while curvature involves a more complex relationship incorporating both first and second derivatives. Matić and Gomez-Marin show this differing relationship to the underlying measurements provides a less inflated basis for assessing the power law relationship, versus the angular velocity formulation’s explicit correlation through radius terms on both sides of the equation. Note, that results presented in the form of the tangential velocity formulation (Eq. 5) may still suffer from correlation. This arises due to the propagation of uncertainty, when tangential velocity is derived from the angular velocity first i.e., $v= \omega /r,$ reintroducing an *r* term. We therefore recommend that power law values are calculated directly from tangential velocity formulation. If it is necessary to report angular velocity results (e.g., for direct comparison), the results of the angular velocity formulation should include a discussion of the concerns mentioned here.

## Concerns with log projection and linear regression in power law calculation

The one-third power law is often derived using linear regression functions, such as MATLAB’s *regress* function*.* However, there is growing awareness of the limitations of linear regression for power law analysis (Clauset et al. 2009). We highlight two specific issues, one concerning y-axis intercept and the influence of measurement uncertainty. Linear regression functions, such as MATLAB’s *regress*, by default force the slope of the regression line to intercept the origin. This presupposes a VGF of one (i.e., ${e}^{0}$, after de-transforming from the log space), thus resulting in an altered signature slope and biased power law exponent values. Thus, at a bare minimum, a linear regression method must be employed in which intercept of the y-axis is permitted. Further, in regression of the one-third power law, there is measurement uncertainty (e.g., noise introduced by recording equipment sensors, quantisation noise and intrinsic motor noise) in both velocity and curvature as they are ultimately derived from coordinate measures. Standard linear regression assumes that the independent variable, the x-axis, in this case the log of curvature $\kappa$, is free from uncertainty (Montgomery et al. 2013, p. 511). Though reliable results can emerge if the error in the dependent variable is larger in magnitude than the independent (Mikkonen et al. 2019; Tellinghuisen 2020), this is untested with respect to power law calculations. York regression (York et al. 2004) accounts for uncertainty in both the dependent and independent variable, but as it lacks a first party implementation available in MATLAB, we do not entertain it as a candidate for the vetted protocol here.

## Alternatives to log projection and linear regression in power law calculation

Even with a trusted York regression implementation, criticisms surround the log transformations necessary to permit any form of linear regression. There is increasing awareness that log-transformation may distort relationships away from those found in untransformed data (Feng et al. 2014). Schaal & Sternad (2001) for instance, have demonstrated that undertaking linear regression, after log–log transformation, methodically minimises deviations from the one-third value. This may be due to the log transformed data underrepresenting errors in high tangential velocities, or the reflect the untested assumption of post transformation normality of errors, potentially violating yet another linear regression assumption (Schaal and Sternad 2001; Zago et al. 2018). Therefore, we highly recommend that regression is conducted on untransformed data. One may then employ non-linear regression functions, such as Levenberg–Marquardt least-squares (LMLS; Schaal and Sternad 2001), or robust methods such as Iteratively Reweighted Least Squares (IRLS; Holland and Welsch 1977) which have immediately accessible implementations.

To summarise, we note that the power law exponent values can be biased by the method of regression that is employed in the calculation of the power law *β* value. This is because linear regression methods are subject to issues surrounding the y-axis intercept, the influence of measurement uncertainty, and need for log-transformed data. We recommend linear regression only to seed the initial values of non-linear least squares regressions, which will in turn offer a more principled path to the power law exponent. We propose LMLS and IRLS non-linear regressions as candidates for the vetted protocol, to replace legacy log projection and linear regression. We implement recommended non-linear regressions below (see Candidate velocity-curvature power law protocol implementation).

## Theoretical perspectives on the VGF’s constancy

The VGF is often taken to be constant. Standard linear regression models implicitly assume this log VGF is a fixed constant across the entire movement trajectory. This mathematical formalization introduces a structural constraint that forces the regression to find a single VGF value that minimizes squared error across all data points, thereby assuming constancy by methodological design rather than empirical verification. However, it has been proposed (Marken and Shaffer 2017) that the VGF is only truly constant for elliptical movements, while other movement patterns exhibit variable VGF values. Whilst there are powerful arguments for scale invariance (Huh and Sejnowski 2015), several studies have documented variant values for VGF when considering scale (Wann et al. 1988; Sternad and Schaal 1999; Schaal and Sternad 2001). In contrast Zago and colleagues (2018) cite work showing that the VGF is *roughly* constant within the physical constraints of typical drawing movement (Dounskaia 2007).

The constancy, or lack thereof, of VGF has significant implications for power law calculations. Marken and Shaffer (2017) argue the ubiquity of the power law arises from an underspecified regression biasing toward values of one-third, due to this inconstant VGF violating linear regressions assumptions. In turn, Zago and colleagues (2018) counter that power law exponents can deviate from the classic one-third value; as seen in the spectrum of power laws and crawling trajectories of larvae. Suggesting divergent values of the power law may reflect genuine biological phenomena rather than statistical artifacts of variant VGF.

## Empirical observations of the VGF’s constancy

Evidence for the constancy of VGF across movement contexts is mixed, with findings varying based on movement type, scale, and experimental conditions. For instance, the VGF is already known to be only piecewise constant in freeform movement (Lacquaniti et al. 1984; Viviani and Cenzato 1985; Hicheur et al. 2005), with this piecewise nature being exploited to segment movement. However this VGF has also been shown to change amidst continuous movements i.e., such as ellipses with larger perimeters, which should be one segment (Sternad and Schaal 1999). This suggests a VGF led segmentation is not mapping to changes in the underlying generator. Constancy of VGF (and indeed *β*) was not found between segments of sign language production, even within a single word. Prompting suggestions a generalised form of the power, with ranges of *β* and VGF may be useful (Endres et al. 2013). In contrast, recent empirical studies have shown VGF and *β* to be constant between ellipse traces of similar geometry within a trial (Zarandi et al. 2023), when participants’ movements were practiced with a metronome. However, this constancy remains unexplored in the spectrum of power laws (self-paced or otherwise). Ultimately, it seems prudent to report and discuss VGF’s variations alongside any power law analysis.

## Addressing methodological concerns in power law experiments

Recording equipment choices may influence recovered power law *β* values. A critical issue concerns the sample rate of the chosen recording device. Commercial tablets especially may exhibit inconsistencies in movement capture rate, requiring resampling to give a consistent digitisation rate (Hickman et al. 2024). Such resampling by default includes an antialiasing low pass filter, e.g., MATLAB’s *resample*. Given the above noted sensitivity to filtering, this should be avoided. Data with inconsistent sampling rates can be handled algorithmically, but it is simplest to exclude such sections from the analysis. Modern commercial tablet devices also introduce new operating systems (e.g., Android, iOS) between digitiser and the exported data. These mobile operating systems are not tuned for the demands of psychophysics experiments. Even desktop operating systems require dedicated packages such as the PsychoPhysics Toolbox, in tandem with NeuroDebian and the *linux-lowlatency* kernel, to render them as precision experimental devices. There have been several attempts to offer applications for smart devices such as Matić and Gomez-Marin’s (2019) Android application for power law research, which purports access to ‘*raw*’ digitiser data, and iOS’s PsyPad (Turpin et al. 2014) and StimuliApp (Marin-Campos et al. 2021) which advertise themselves as suitable psychophysics environments. Android tablets may sample at 60 Hz or even below, whereas in laboratory settings movements are often recorded with professional draughting equipment, with published accuracy values and consistent sampling rates e.g., 100, 133 or 200 Hz. Hence, systematic validation of lower sampling rates is advisable. This could be undertaken by adopting the 240 Hz digitiser equipped iPad Pro (Marin-Campos et al. 2021) and comparing results at 60, 120 and 240 Hz, by addressing subsets of the data, not by resampling.

## Concerning tempo

A major methodological concern, which pre-dates commercial tablets, is the possible existence of a critical tempo for the emergence of the power law. Whilst tempos from metronomes figure prominently in the literature (e.g., Zago et al. 2018; Zarandi et al. 2023), such metronomes cannot be used for spontaneous freehand drawing, and there is no agreement as to what this tempo should be. There is evidence of a critical tempo above which the power law emerges reliably (Wann et al. 1988; Kuberski and Gafos 2019; Matić 2022). Matić (2022) notes that the one-third power law goodness of fit ${(r}^{2})$ only equals or exceeds three-quarters at tempos exceeding ~ 1 Hz. We speculate that such tempos enforce ‘*fluid*’ motor production, via rhythmicity of movement. Unfortunately, this ~ 1 Hz threshold is not universal, as noted by Matić, it is specific to the ellipse geometries they employed. Wann and colleagues, observe ellipses drawn at 1 Hz (which arose without metronome, from the instruction ‘*easy, comfortable pace*’), were not always consistent with the one-third power law. At a ‘*fast*’ pace (~ 1.5 Hz) Wann and colleagues saw consistent results of roughly one-third. Whereas in human–robot kinematic dyads researchers (West et al. 2022; Edraki et al. 2023) have comfortably employed periods of three seconds i.e., 1/3 Hz. Therefore, for arbitrary ellipses, repeating Matić’s work with a range of geometries is indicated to gain insight into this potential tempo threshold.

In the absence of such work, we advocate for the use of naturalistic movement speeds i.e., the ‘*easy, comfortable pace*’, as suggested by Wann and colleagues (1988), coupled with movement repetitions at an increased pace ‘*approximately twice their previous speed’*. The former comfortable pace will capture naturalistic movement but risks not achieving the threshold for specific ellipse perimeters and eccentricities, whilst the latter, faster movement should exceed any tempo threshold as Wann and colleagues found. The literature does not address whether there is an upper threshold beyond which the power law becomes inconsistent again, only that it becomes inconsistent in one-third compliance or in goodness of fit at lower tempos. Though admittedly subjective, these self-pacing instructions can then be used verbatim with freehand tasks.

For a given geometry, tempo can be derived from the VGF (if constant). However, we advocate reporting VGFs, geometries (i.e., major and minor axes or perimeter and eccentricity) and tempo. Reporting tempos, as well as geometry, is necessary to allow replication and direct comparison with future experiments. For example, research investigating the neural correlates of the one-third power law—by presenting participants with synthetic data and making neural recordings—often does not report the tempo (or the values of VGF, of which it is a function) of the synthetic motion presented (Dayan et al. 2007; Casile et al. 2010; Meirovitch et al. 2015). Hence, neural correlates discerned may be to motion not fully compliant with the power law, i.e., with VGFs in unknown compliance with a naturalistic tempo.

The geometry of the ellipse alone is not sufficient for experimental consistency, as there is evidence of preferred orientation in ellipse drawing (Danna et al. 2011). Danna and colleagues report higher ellipse tracing tempo and accuracy for a range of orientations. Therefore, power law results may differ with variations of tempo, and accuracy impact on the noise envelope. Preferred orientations are found in the range of ellipses whose long axis are aligned along the sagittal axis (i.e. perpendicular to the shoulders), up to an angle of 75° from the sagittal plane. There is diminished trace tempo and accuracy between −90° and −30°. These values are inverted for left handers (i.e. 0° to −75° preferred to 30°–90°). An ellipse with long axis perpendicular to the sagittal plane (i.e. parallel to the shoulders) would fall outside this range for both. Consistency with these preferred values should be sought within, and between experiments. Preferred orientations have not been explored for the spectrum of angular frequency shapes, but given they are also sinusoidal in *x* and *y* we speculate they will exhibit similar preferred drawing orientations. However free hand drawings will not likely be impacted by the orientation of the canvas.

## Limitations of this review

This review considers findings from diverse research methodologies that differ in recording apparatus—ranging from 3D motion capture and unconstrained gestures to stylus or finger input on an instrumented surface—reflecting distinct underlying movements, potentially recruiting some or all of wrist, elbow, and shoulder in addition to fingers. The paradigms also vary in geometry, such as guidance via a cut-out template or tracing a displayed image, which may be progressively revealed or entirely concealed using a blindfold. Additionally, they differ in tempo, whether enforced by a metronome, merely practiced with one, spontaneous, or guided by subjective command (e.g., fast or at a comfortable pace). This methodological heterogeneity may undermine the applicability of recommendations to non-matching methodologies. Future research should systematically examine how these methodological choices impact power law calculations.

## Candidate velocity-curvature power law protocol implementation

In the previous sections we have reported suggestions from the literature that the one-third velocity-curvature power law calculation is sensitive to legacy analysis choices including filtering, differentiation, log-transformation, and linear regression. Such steps may mask, distort, or spuriously confirm the power law. We proposed candidate protocol elements drawn from the literature that should minimise the issues observed in the legacy protocol. In the following section we consider the results of both legacy and candidate protocols elements in combination, in modular open science functions. We then evaluate the legacy and candidate elements and suggest a ‘vetted’ velocity curvature power law analysis protocol. There are two main criteria that an element must pass to be included in the vetted protocol. Firstly, results must exclude spurious one-third power law confirmations, such as when synthetic data is generated with *β* = 0 as discussed above. Secondly, to be considered vetted, any protocol must produce comparable *β* values in paradigms in which the one-third power law is anticipated, such as ellipse traces with minimal noise. As an additional practical consideration, we consider only analysis elements with existing support in the literature, and professional implementations in MATLAB.

In the following section we introduce the results of two scripts. These scripts demonstrate that the candidate elements, together in a protocol, alone meet these two criteria, when considered with simulated and freely available empirical data. The scripts and underlying functions are available at this paper’s GitHub repository (https://github.com/dagmarfraser/velocity-curvature-power-law-protocol).

## Comparing the candidate and legacy protocol implementations on synthetic data

**Fig. 5:**
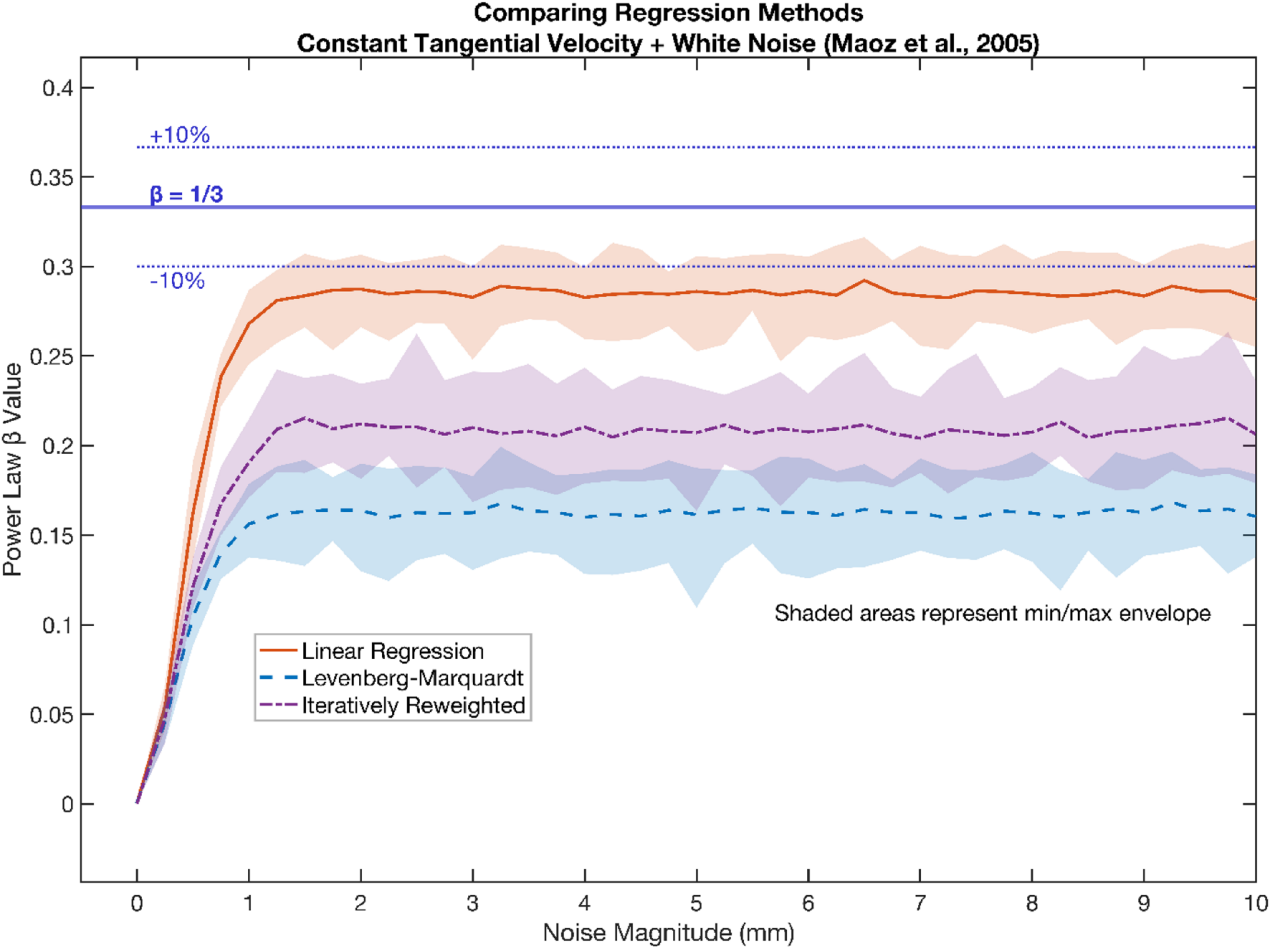
Shows *β* power law exponents (y-axis) obtained via; linear legacy (red line), and candidate Levenberg–Marquardt (blue dash), and candidate iteratively reweighted (purple dot dash) least square regressions. Synthetic elliptical trajectories (with 100 mm major axis and 50 mm minor axis), and tempo 1 Hz, were generated with a *β* of zero (i.e. constant tangential velocity), and then admixed with increasing standard deviations (mm) of Gaussian white noise (x-axis), generating N = 30 trajectories at each noise magnitude. These trajectories where then sampled at 100 Hz. As noise increases all regression methods produce *β*’s that deviate from the expected zero value. Notably, in the case of the standard linear regression (with intercept, implemented here via MATLAB’s *fitlm*) individual values approximate a one-third power law at higher noise magnitudes, as previously observed (Maoz et al. 2005). A spurious one-third confirmation is not observed in candidate regressions Levenberg–Marquardt, or iteratively reweighted least squares

**Fig. 6:**
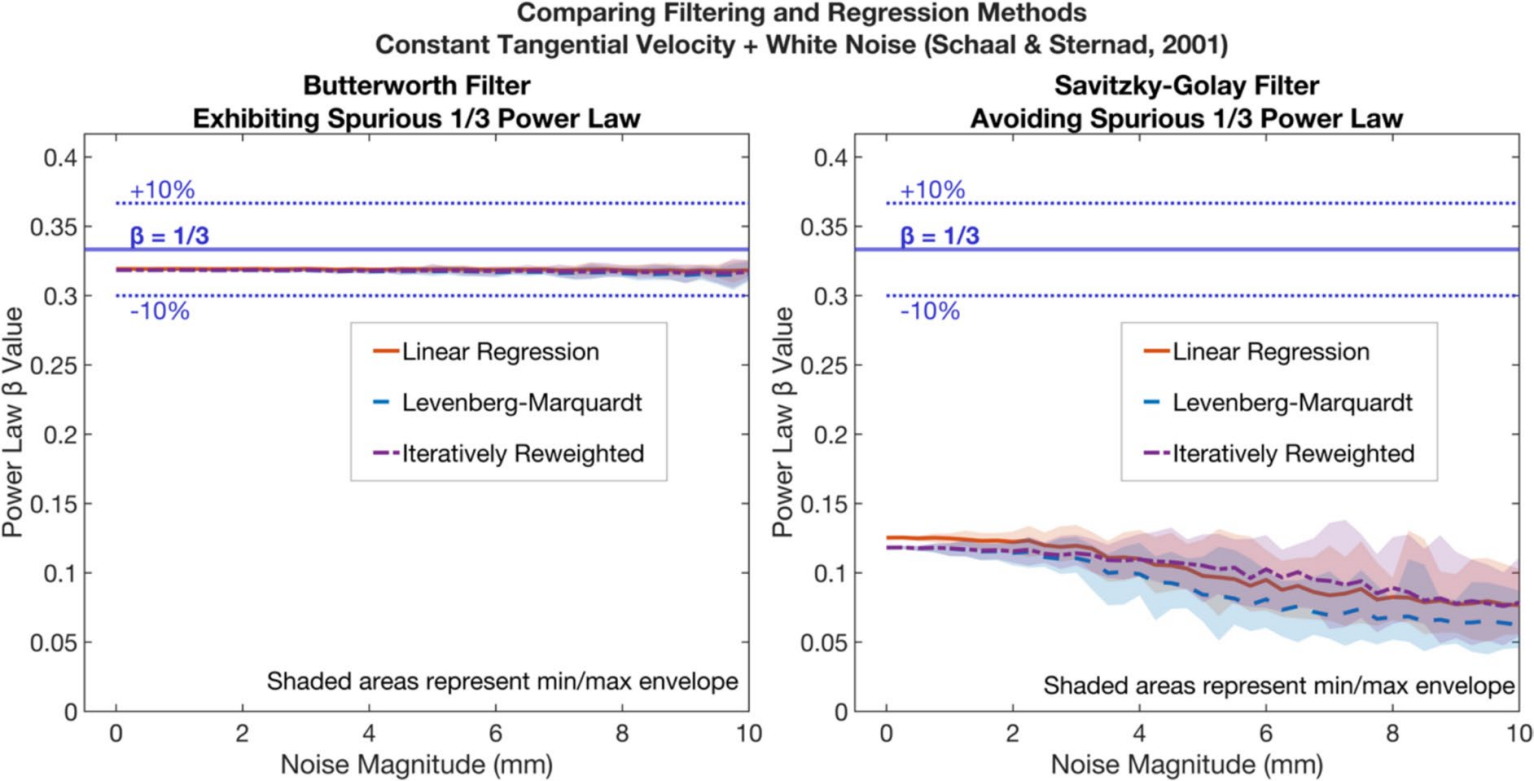
Plots of the *β* power law exponents (y-axis) for three types of regression; legacy linear (red line), candidate Levenberg–Marquardt (blue dash), and candidate iteratively reweighted (purple dot dash) least square regressions. Using data processed via (left panel) legacy finite differences differentiation and Butterworth 4th order low pass filtering, or via (right panel) candidate Savitzky-Golay differential filtering. Synthetic elliptical trajectories (with 200 mm major axis and 100 mm minor axis) generated with constant tangential velocity, and tempo of 1.667 Hz, were admixed with increasing standard deviations (mm) of Gaussian white noise (x-axis), generating N = 30 trajectories at each noise magnitude. These trajectories were then sampled at 100 Hz. The left panel’s legacy Butterworth filtering, at zero noise, aligns with prior results (Schaal and Sternad 2001), in which authors noted the spurious emergence of a one-third power law. Though the right panel candidate SG filtered data demonstrates a non-zero result, it does not spuriously confirm the one-third power law

To address the first criteria (i.e., no spurious confirmation of the one-third power law) we share a script, *PowerLawSynthetic.m* implementing candidate and legacy protocol elements to examine synthetic elliptical trajectories generated from detailed descriptions by Maoz and colleagues (2005, see Fig. 5) and Schaal and Sternad (2001, see Fig. 6). In the case of Maoz and colleagues (2005) to demonstrate the effect of noise, and in Schaal and Sternad (2001) the effects of 4^th^ order Butterworth filtering. Both papers employ synthetic elliptical trajectories (i.e., angular frequency *φ* of two), generated by setting *β* to zero. The calculated *β* from such trajectories should be zero if the analysis is unbiased. Consider Eq. (10), if the *β* exponent is set to zero in the classic power law formulation, the curvature term ${\kappa }^{0}$ is therefore one. The purportedly constant VGF, thus gives rise to a constant tangential velocity $v$, equal to VGF*.*

10 $$v = VGF {\kappa }^{0}$$

In Fig. 5 we confirm the result of Maoz and colleagues (2001), showing legacy linear regression of noise admixed trajectories offers artificial confirmation of a roughly one-third power law. We further demonstrate that spurious confirmatory one-third values do not arise when employing the candidate choices of LMLS or IRLS regression regardless of noise. In Fig. 6 we confirm the result of Schaal and Sternad (2001) that artificial confirmation of one-third occurs after legacy 4^th^ order Butterworth filtering. We further demonstrate that this does not occur when using candidate SG smoothing differential filters as shown in Fig. 6, regardless of the ensuing regression choices. Thus, only the candidate protocol elements, i.e., non-linear regressions (LMLS or IRLS) and SG filtering, meet criteria one (avoiding spurious one-third power law compatible results in constant tangential velocity synthetic ellipse data).

It should be noted that in neither Fig. 5 nor the right panel of Fig. 6 is the true *β* of zero recovered, only that the spurious one-third is avoided. In Fig. 5 we see plateaus suggesting that each regression has a natural value for calculation when dominated by white Gaussian noise. Figure 5 suggests that adding white Gaussian noise, of standard deviation ~ 2 mm is enough to bias results away from the underlying true exponent of zero, to these plateaus.

In the left panel of Fig. 6, a signal generated with a *β* of zero, generates a spurious *β* of roughly one-third. In filtering the noise, the Butterworth filtering has also altered the underlying signal. Given this propensity of the legacy 4^th^ order Butterworth filter to give rise to *β* of one-third, that filter must be avoided in the analysis. In contrast, the candidate SG smoothly differentiated regressions (Fig. 6’s right panel) avoids spurious one-third confirmation. Therefore, only the candidate SG (with any regression) passes the first criteria.

As Crenna and colleagues (2021) designed SG filter parameters to match the Butterworth, the difference between Fig. 6’s Butterworth and SG panels’ results merits examination. The Butterworth regressions of Fig. 6’s left panel give near identical results at all noise intensities, suggesting there little remaining unfiltered noise. The absence of noise eliminates concerns with error normality in log projection (non-linear vs linear regressions) and outliers (to which only iteratively reweighted least squares is robust). Whilst removing ‘*noise*’ is desirable, in this case the filter alters the underlying signal, biasing the recovered *β* towards one-third. This aligns with suggestions (Gribble and Ostry 1996; Schaal and Sternad 2001) that powerful smoothing inevitably gives rise to the power law in rhythmic motion, which is coincident with the maximal smoothness minimum jerk neural schema of Viviani and Flash (1995). Figure 6’s right panel shows the SG retains noise in the envelopes of *β*, as the regression differ. This lesser degradation of peaks is one of the key advantages of the SG versus other filtering schemes (Savitzky and Golay 1964), retaining fine structure of individual differences in trajectories, otherwise lost with the Butterworth filter.

## Comparing the candidate and legacy protocol implementations on empirical data

**Fig. 7:**
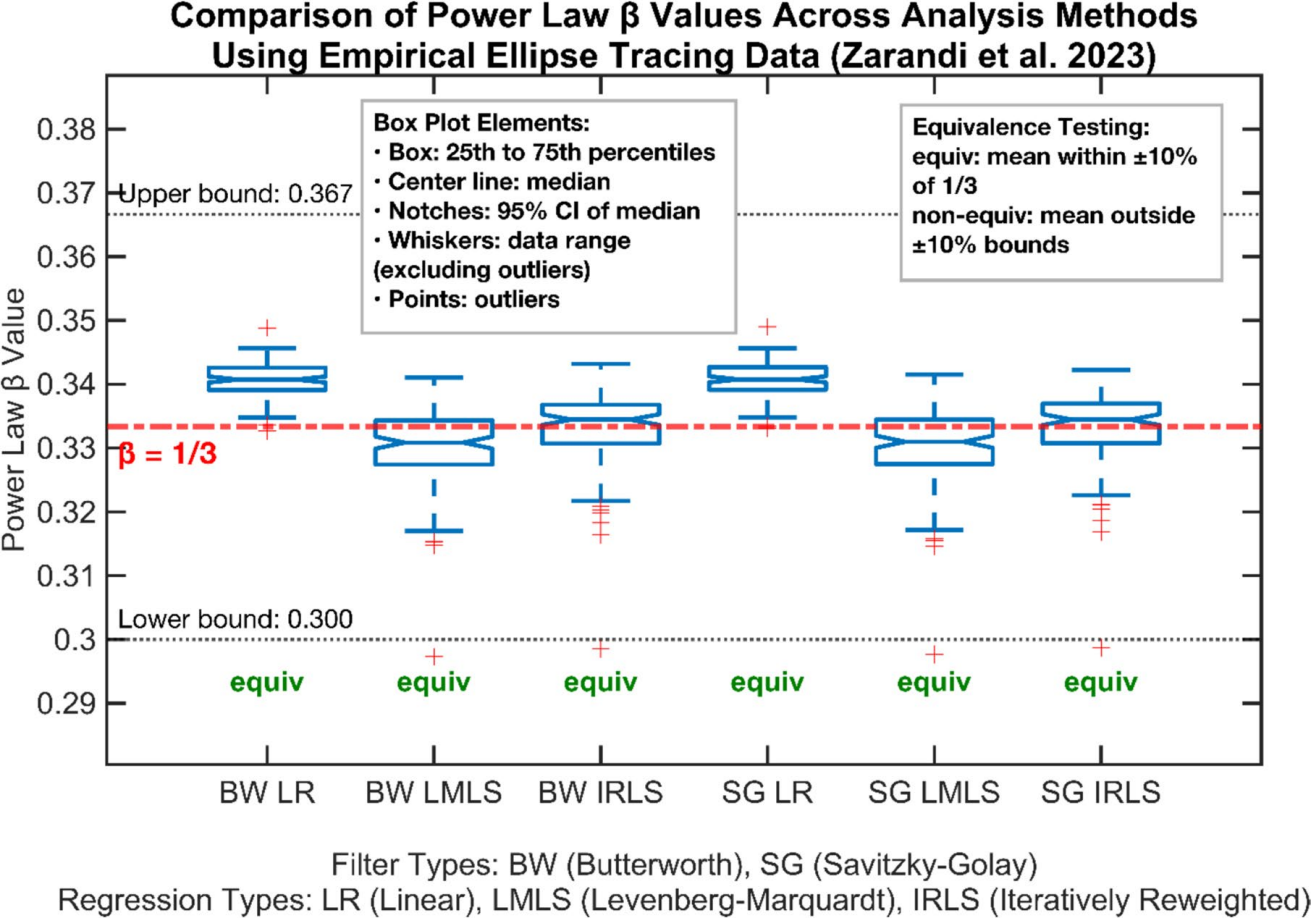
Boxplots of power law exponents *β* (y-axis) employing empirical data (N = 14) from Zarandi et al. (2023) at 100 Hz. Human participants traced ellipses on a WACOM Bamboo Slate; in ‘*optimal*’ conditions in which the one-third power law should arise i.e., angular frequency *φ* of two, with 100 mm major and 20 mm minor axis, 45° inclination, practiced with greater than 1 Hz metronome. Data exhibited limited noise (eccentricity differed between template and trajectory by less than 1%) and constant Velocity Gain Factor *within* trial, i.e., per perimeter. Power law exponents are calculated using all available combinations of legacy and candidate methods; finite differences differentiation and Butterworth 4^th^ order low order filtering (left three), or via Savitzky-Golay 4^th^ order differential smoothing (right three). Followed by either legacy linear regression, candidate Levenberg–Marquardt or candidate iteratively reweighted least squares regressions. Methods labelled ‘equiv’ have means falling within ± 10% of *β* = 1/3 (p < 0.05), demonstrating statistical equivalence to the theoretical power law exponent

A second script, *PowerLawEmpirical.m* deploys candidate and legacy protocols, to calculate power law *β*’s for human participant ellipses (Zarandi et al. 2023) in which we should anticipate one-third. The results in Fig. 7 demonstrate these anticipated *β*’s of roughly one-third. Values between the left three legacy Butterworth filtered and the rightward candidate SG groups were examined with Two One-Sided Tests, which suggest all method combinations give results equivalent to one-third ± 10%, i.e., values that would normally be taken in the literature as support of the roughly one-third value. Therefore, the anticipated one-third value emerges from both the legacy and candidate protocol non-linear regressions, thus they both pass the second criteria. This suggests that Crenna and colleagues (2021) SG filter accurately mimics the desirable elements of Butterworth filtering in this instance. This is in stark contrast to Fig. 6, in which the Butterworth filter provides spurious one-third confirmation failing criteria one, which the SG avoids. This re-affirms the candidate SG as a suitable replacement for the Butterworth and finite differences differentiation.

## Legacy and candidate protocols contrasted

Considering the two criteria, we note the legacy protocol only satisfies the second criteria: reporting roughly one-third values in empirical data expected to have to have a *β* of one-third. The legacy protocol fails the first criteria, i.e., it does not avoid spurious confirmation of a one-third power law in *synthetic* data with a generative *β* of zero. Therefore, when employing the legacy protocol, experimenters cannot be certain that any one-third value reported is due to the underlying data, noise characteristics, filtering employed*,* log projection or linear regression biases. In contrast, the candidate protocol of SG and non-linear regressions meets both criteria. We therefore recommend adopting the candidate protocol as the vetted protocol. Under such a protocol there is increased certitude that any one-third value recovered reflects an intrinsic value of the generator rather than biases introduced by analysis choices.

However, whilst this vetted protocol is demonstrably superior, concerns remain. In Fig. 5 non-linear regressions plateau at nonzero values (i.e., not recovering zero) in the presence of noise. In Fig. 6, nonzero *β* values arise with no noise, suggesting the SG filtering also biases the result, even if it does not artificially confirm the one-third value. However, equipped with these values, experimenters can identify suspect results dominated by noise, when they exhibit these values.

## Summary and recommendations

In summation, this review demonstrates a more principled, vetted path to power law calculation. Within the review we show that only analyses employing SG smoothing differential filter and either LMLS or IRLS non-linear regressions meet two criteria of usefulness. Firstly, artificial one-third confirmations are avoided, and secondly anticipated one-third results are robustly reproduced. Legacy calculations fail the first criteria. This vetted protocol is offered in an open science (https://github.com/dagmarfraser/velocity-curvature-power-law-protocol), modular implementation. It is hoped this repository will speed adoption and permit the field to add further candidate practices as they emerge and generate comparative results. The vetted protocol’s application to the spectrum of power laws, and to freehand motion remains unexplored. However, there is confidence that ensuing results of this would escape the one-third confirmatory biases of prior analysis protocols.

## Data Availability

The data and vetted analysis protocol arising from the review is available in the *velocity-curvature-power-law-protocol* repository, which can be found here https://github.com/dagmarfraser/velocity-curvature-power-law-protocol.

## Footnotes

1 The constant velocity gain factor (VGF), was explored in a companion paper by Lacquaniti and colleagues (1984), but is often omitted in the literature (e.g., as in Eq. 1, and in Figs. 1 and 2).

2 Whilst Schaal and Sternad’s low pass cut-off frequency of 2.5 Hz is lower than common in the field, it is of the same magnitude of the widely used 10 Hz, and exceeds 0.07 Hz found in recent papers (Zago et al. 2016; Zarandi et al. 2023).
